# 4,4′-Dimethyl-2,2′-[imidazolidine-1,3-diylbis(methyl­ene)]diphenol

**DOI:** 10.1107/S1600536812042808

**Published:** 2012-10-20

**Authors:** Augusto Rivera, Luz Stella Nerio, Jaime Ríos-Motta, Monika Kučeraková, Michal Dušek

**Affiliations:** a Universidad Nacional de Colombia, Sede Bogotá, Facultad de Ciencias, Departamento de Química, Cra 30 No.45-03, Bogotá, Código Postal 111321, Colombia; b Institute of Physics ASCR, v.v.i., Na Slovance 2, 182 21 Praha 8, Czech Republic

## Abstract

The imidazolidine ring in the title compound, C_19_H_24_N_2_O_2_, adopts a twist conformation and its mean plane (r.m.s. deviation = 0.19 Å) makes dihedral angles of 72.38 (9) and 71.64 (9)° with the two pendant aromatic rings. The dihedral angle between the phenyl rings is 55.94 (8)°. The mol­ecular structure shows the presence of two intra­molecular O—H⋯N hydrogen bonds between the phenolic hydroxyl groups and N atoms with graph-set motif *S*(6). In the crystal, C—H⋯O hydrogen bonds lead to the formation of chains along the *b-*axis direction.

## Related literature

For the anti-inflammatory and analgesic properties of imidazolidines, see: Sharma & Khan (2001 ▶). For related structures, see: Rivera *et al.* (2011 ▶, 2012 ▶). For the preparation of the title compound, see: Rivera *et al.* (1993 ▶). For standard bond lengths, see: Allen *et al.* (1987 ▶). For ring conformations, see Cremer & Pople (1975 ▶). For hydrogen-bond graph-set nomenclature, see: Bernstein *et al.* (1995 ▶).
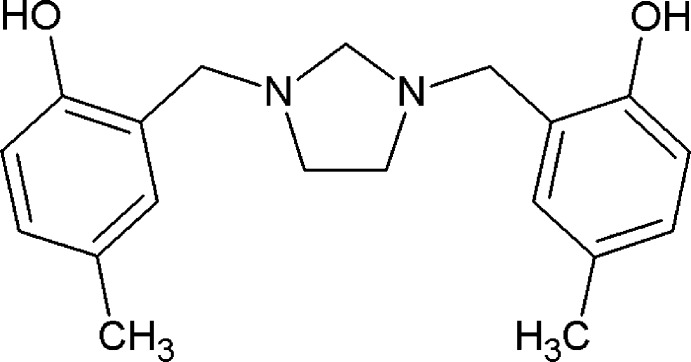


## Experimental

### 

#### Crystal data

C_19_H_24_N_2_O_2_
*M* *_r_* = 312.4
Monoclinic, 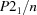

*a* = 11.5029 (4) Å
*b* = 9.5001 (3) Å
*c* = 16.1874 (6) Å
β = 107.078 (3)°
*V* = 1690.94 (10) Å^3^
*Z* = 4
Cu *K*α radiation
μ = 0.63 mm^−1^
*T* = 120 K
0.25 × 0.22 × 0.13 mm

#### Data collection

Agilent Xcalibur Atlas Gemini ultra diffractometer
Absorption correction: multi-scan (*CrysAlis PRO*; Agilent, 2010 ▶) *T*_min_ = 0.573, *T*_max_ = 1
12982 measured reflections
3007 independent reflections
2648 reflections with *I* > 3σ(*I*)
*R*_int_ = 0.021

#### Refinement

*R*[*F* ^2^ > 2σ(*F* ^2^)] = 0.033
*wR*(*F* ^2^) = 0.104
*S* = 1.93
3007 reflections
215 parameters
H atoms treated by a mixture of independent and constrained refinement
Δρ_max_ = 0.16 e Å^−3^
Δρ_min_ = −0.14 e Å^−3^

### 

Data collection: *CrysAlis PRO* (Agilent, 2010 ▶); cell refinement: *CrysAlis PRO*; data reduction: *CrysAlis PRO*; program(s) used to solve structure: *Superflip* (Palatinus & Chapuis 2007 ▶); program(s) used to refine structure: *JANA2006* (Petříček *et al.*, 2006 ▶); molecular graphics: *DIAMOND* (Brandenburg & Putz, 2005 ▶); software used to prepare material for publication: *JANA2006*.

## Supplementary Material

Crystal structure: contains datablock(s) global, I. DOI: 10.1107/S1600536812042808/lr2083sup1.cif

Structure factors: contains datablock(s) I. DOI: 10.1107/S1600536812042808/lr2083Isup2.hkl

Supplementary material file. DOI: 10.1107/S1600536812042808/lr2083Isup3.cml

Additional supplementary materials:  crystallographic information; 3D view; checkCIF report

## Figures and Tables

**Table 1:** Hydrogen-bond geometry (Å, °)

*D*—H⋯*A*	*D*—H	H⋯*A*	*D*⋯*A*	*D*—H⋯*A*
O1—H2⋯N2	0.912 (17)	1.869 (16)	2.6893 (13)	148.6 (16)
O2—H1⋯N1	0.923 (17)	1.825 (15)	2.6807 (12)	153.0 (15)
C17—H1*c*17⋯O1^i^	0.96	2.48	3.4286 (14)	168.38

Symmetry code: (i) 
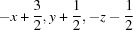
.

**Fig. 1.**
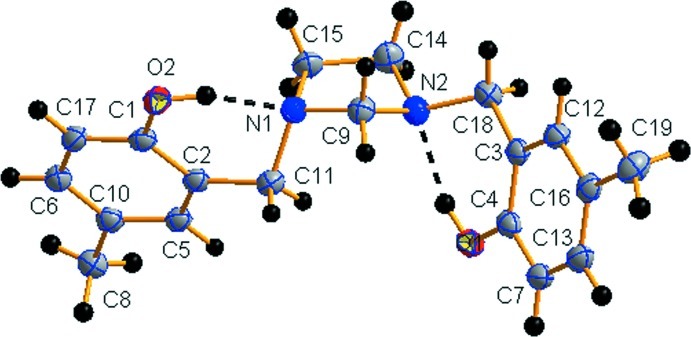
A perspective view of the title compound. Displacement ellipsoids are drawn at the 50% probability level and H atoms are shown as small spheres of arbitrary radii. Hydrogen bonds are drawn as dashed lines.

**Fig. 2.**
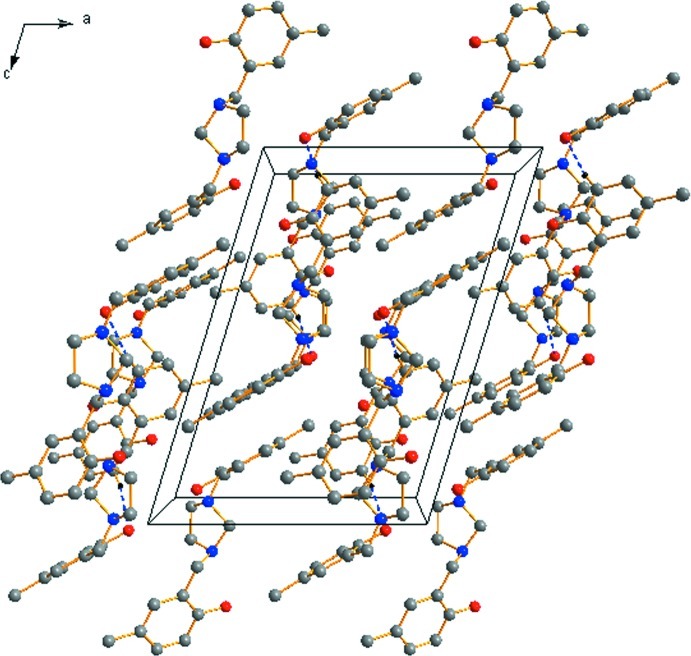
Packing of the molecules of the title compound view along b axis.

## supplementary crystallographic information

### Comment

The 1,3-imidazolidine system is intriguing because it is present in biologically active molecules with anti-inflammatory and analgesic properties (Sharma *et al.*, 2001). In our current investigations of factors which influence intramolecular hydrogen bond strength in 1,3-imidazolidine-bridged bis(phenols) (Rivera *et al.*, 2011, 2012), we turn our attention to title compound (I) because the methyl substituent at the *para*-position in aromatic rings is an electron-donating group which makes the negative charge of hydroxyl group.

The molecular structure and atom-numbering scheme for (I) are shown in Fig. 1 The imidazolidine ring adopts a twist conformation, with twist about the C9—N2 bond; the puckering parameters (Cremer & Pople, 1975), Q2 = 0.4008 (13) Å and φ2 = 51.81 (18)°. Intraanular bond lengths (Allen *et al.*, 1987) and angles of (I) are within normal ranges and are comparable to similar structures (Rivera *et al.*, 2011, 2012). The mean plane of imidazolidine ring defined by N1, C15 and C14 makes a dihedral angle of 72.375 (85)° and 71.644 (96)° with the two pendant aromatic rings, C1/C2/C5/C10/C6/C17 and C3/C4/C7/C13/C16/C12 respectively. The dihedral angle between the phenyl rings is 55.938 (83)°. Its X-ray structure confirms the presence of intramolecular hydrogen bonds between the phenolic hydroxyl groups and nitrogen atoms with graph-set motif S(6) (Bernstein *et al.*, 1995) (Table 1). The observed N···O distances [2.6807 (12) Å and 2.6893 (13) Å] and the observed C–O bond lengths [1.3701 (16) Å and 1.3715 (15) Å] are longer in relation to the unsubstituted related structures [2.6557 (13) Å and 1.3654 (15) Å, respectively] (Rivera *et al.*, 2012) and *p*-chloro derivative [2.6524 (17) Å and 1.366 (2) Å, respectively] (Rivera *et al.*, 2011). This result could indicate that the electro-donating nature of the methyl group at *para*-position influences the strength of the intra-molecular hydrogen bond.

In the crystal, intermolecular C—H···O hydrogen bonds lead to the formation of chains along the *b* axis, (Table 1, Fig. 2).

### Experimental

For the originally reported synthesis, see: Rivera *et al.* (1993)

### Refinement

The position of hydrogen atoms attached to carbon were fixed in geometrically expected positions, with C—H distance 0.96 Å. On the other hand, positions of H atoms of OH groups were refined without any restrain or constrain. ADP of all hydrogen atoms were fixed as 1.2 multiple of the equivalent isotropic ADP of their parent atom

### Crystal data

**Table:**

C_19_H_24_N_2_O_2_	*F*(000) = 672
*M**_r_* = 312.4	*D*_x_ = 1.227 Mg m^−^^3^
Monoclinic, *P*2_1_/*n*	Cu *K*α radiation, λ = 1.5418 Å
Hall symbol: -P 2yn	Cell parameters from 8356 reflections
*a* = 11.5029 (4) Å	θ = 4.0–66.9°
*b* = 9.5001 (3) Å	µ = 0.63 mm^−^^1^
*c* = 16.1874 (6) Å	*T* = 120 K
β = 107.078 (3)°	Pyramidal shape, white
*V* = 1690.94 (10) Å^3^	0.25 × 0.22 × 0.13 mm
*Z* = 4	

### Data collection

**Table:**

Agilent Xcalibur Atlas Gemini ultra diffractometer	3007 independent reflections
Radiation source: Enhance Ultra (Cu) X-ray source	2648 reflections with *I* > 3σ(*I*)
Mirror monochromator	*R*_int_ = 0.021
Detector resolution: 10.3784 pixels mm^-1^	θ_max_ = 67.1°, θ_min_ = 4.2°
ω scans	*h* = −13→12
Absorption correction: multi-scan (*CrysAlis PRO*; Agilent, 2010)	*k* = −11→11
*T*_min_ = 0.573, *T*_max_ = 1	*l* = −19→18
12982 measured reflections	

### Refinement

**Table:**

Refinement on *F*^2^	H atoms treated by a mixture of independent and constrained refinement
*R*[*F*^2^ > 2σ(*F*^2^)] = 0.033	Weighting scheme based on measured s.u.'s *w* = 1/(σ^2^(*I*) + 0.0016*I*^2^)
*wR*(*F*^2^) = 0.104	(Δ/σ)_max_ = 0.0004
*S* = 1.93	Δρ_max_ = 0.16 e Å^−^^3^
3007 reflections	Δρ_min_ = −0.14 e Å^−^^3^
215 parameters	Extinction correction: B-C type 1 Gaussian isotropic (Becker & Coppens, 1974)
0 restraints	Extinction coefficient: 1300 (400)
90 constraints	

### Special details

**Table:**

Experimental. Empirical absorption correction using spherical harmonics, implemented in SCALE3 ABSPACK scaling algorithm.
Refinement. The refinement was carried out against all reflections. The conventional *R*-factor is always based on *F*. The goodness of fit as well as the weighted *R*-factor are based on *F* and *F*^2^ for refinement carried out on *F* and *F*^2^, respectively. The threshold expression is used only for calculating *R*-factors *etc*. and it is not relevant to the choice of reflections for refinement.The program used for refinement, Jana2006, uses the weighting scheme based on the experimental expectations, see _refine_ls_weighting_details, that does not force *S* to be one. Therefore the values of *S* are usually larger than the ones from the *SHELX* program.

### Fractional atomic coordinates and isotropic or equivalent isotropic displacement parameters (Å^2^)

**Table:**

	*x*	*y*	*z*	*U*_iso_*/*U*_eq_	
O1	0.88856 (7)	0.44965 (9)	0.06241 (5)	0.0310 (3)	
O2	0.64693 (7)	0.76997 (9)	−0.30769 (6)	0.0290 (3)	
N1	0.76722 (9)	0.73615 (10)	−0.13993 (6)	0.0266 (3)	
N2	0.84361 (8)	0.71755 (10)	0.00889 (6)	0.0261 (3)	
C1	0.74585 (10)	0.70626 (11)	−0.32261 (7)	0.0251 (3)	
C2	0.82536 (10)	0.62323 (11)	−0.25869 (7)	0.0243 (3)	
C3	0.76676 (10)	0.60644 (12)	0.12013 (7)	0.0247 (3)	
C4	0.80273 (10)	0.47037 (12)	0.10465 (7)	0.0267 (4)	
C5	0.92398 (10)	0.56062 (11)	−0.27747 (7)	0.0245 (3)	
C6	0.86516 (11)	0.66039 (12)	−0.41913 (7)	0.0290 (4)	
C7	0.75212 (11)	0.35433 (13)	0.13319 (8)	0.0313 (4)	
C8	1.05237 (11)	0.50485 (13)	−0.37565 (8)	0.0305 (4)	
C9	0.72950 (10)	0.71770 (12)	−0.06231 (7)	0.0273 (4)	
C10	0.94551 (10)	0.57626 (12)	−0.35724 (7)	0.0261 (4)	
C11	0.80246 (10)	0.60126 (12)	−0.17246 (7)	0.0256 (4)	
C12	0.67858 (10)	0.62103 (12)	0.16280 (7)	0.0262 (4)	
C13	0.66608 (11)	0.37235 (13)	0.17686 (8)	0.0315 (4)	
C14	0.91127 (11)	0.83498 (14)	−0.01388 (8)	0.0336 (4)	
C15	0.87305 (12)	0.83398 (13)	−0.11242 (8)	0.0335 (4)	
C16	0.62725 (10)	0.50583 (12)	0.19224 (7)	0.0280 (4)	
C17	0.76650 (11)	0.72510 (12)	−0.40199 (8)	0.0283 (4)	
C18	0.82742 (10)	0.73307 (12)	0.09490 (7)	0.0272 (4)	
C19	0.53567 (12)	0.52729 (15)	0.24135 (9)	0.0374 (4)	
H1c5	0.979186	0.504614	−0.233853	0.0294*	
H1c6	0.878406	0.67367	−0.474459	0.0348*	
H1c7	0.776818	0.261183	0.122626	0.0375*	
H1c8	1.072127	0.552979	−0.421921	0.0366*	
H2c8	1.121122	0.507333	−0.324784	0.0366*	
H3c8	1.031868	0.408791	−0.391977	0.0366*	
H1c9	0.689835	0.628276	−0.064525	0.0327*	
H2c9	0.680476	0.796361	−0.055893	0.0327*	
H1c11	0.738473	0.533616	−0.178617	0.0307*	
H2c11	0.874835	0.565476	−0.131679	0.0307*	
H1c12	0.652179	0.713903	0.172286	0.0315*	
H1c13	0.632626	0.291154	0.196901	0.0378*	
H1c14	0.996946	0.816774	0.007957	0.0403*	
H2c14	0.886032	0.921709	0.006006	0.0403*	
H1c15	0.848056	0.926862	−0.133682	0.0402*	
H2c15	0.938576	0.798444	−0.131927	0.0402*	
H1c17	0.712548	0.782964	−0.445198	0.034*	
H1c18	0.77967	0.81553	0.09603	0.0327*	
H2c18	0.905134	0.747643	0.136757	0.0327*	
H1c19	0.480459	0.601158	0.21462	0.0449*	
H2c19	0.491052	0.441692	0.240675	0.0449*	
H3c19	0.577222	0.552689	0.300002	0.0449*	
H1	0.6668 (13)	0.7721 (15)	−0.2481 (11)	0.0348*	
H2	0.8974 (14)	0.5333 (17)	0.0373 (10)	0.0372*	

### Atomic displacement parameters (Å^2^)

**Table:**

	*U*^11^	*U*^22^	*U*^33^	*U*^12^	*U*^13^	*U*^23^
O1	0.0325 (5)	0.0315 (5)	0.0289 (4)	0.0074 (3)	0.0088 (3)	−0.0025 (3)
O2	0.0265 (4)	0.0304 (5)	0.0291 (5)	0.0048 (3)	0.0066 (3)	0.0029 (3)
N1	0.0280 (5)	0.0255 (5)	0.0267 (5)	0.0009 (4)	0.0087 (4)	0.0016 (4)
N2	0.0238 (5)	0.0292 (5)	0.0253 (5)	−0.0011 (4)	0.0073 (4)	−0.0005 (4)
C1	0.0243 (6)	0.0208 (5)	0.0284 (6)	−0.0011 (4)	0.0050 (4)	−0.0012 (4)
C2	0.0246 (5)	0.0209 (5)	0.0257 (6)	−0.0031 (4)	0.0048 (4)	0.0003 (4)
C3	0.0251 (5)	0.0261 (6)	0.0203 (5)	0.0005 (4)	0.0026 (4)	−0.0016 (4)
C4	0.0262 (6)	0.0287 (6)	0.0216 (5)	0.0036 (4)	0.0016 (4)	−0.0029 (4)
C5	0.0243 (5)	0.0202 (5)	0.0265 (6)	−0.0012 (4)	0.0034 (4)	0.0007 (4)
C6	0.0345 (6)	0.0273 (6)	0.0248 (6)	−0.0022 (5)	0.0081 (5)	−0.0005 (4)
C7	0.0379 (7)	0.0239 (6)	0.0273 (6)	0.0035 (5)	0.0023 (5)	−0.0017 (5)
C8	0.0311 (6)	0.0295 (6)	0.0311 (6)	−0.0002 (5)	0.0096 (5)	−0.0028 (5)
C9	0.0244 (6)	0.0298 (6)	0.0276 (6)	0.0021 (4)	0.0075 (5)	0.0014 (4)
C10	0.0268 (6)	0.0224 (5)	0.0281 (6)	−0.0032 (4)	0.0065 (4)	−0.0020 (4)
C11	0.0246 (5)	0.0239 (5)	0.0277 (6)	0.0007 (4)	0.0070 (4)	0.0029 (4)
C12	0.0265 (6)	0.0245 (6)	0.0260 (5)	0.0025 (4)	0.0050 (4)	−0.0004 (4)
C13	0.0356 (6)	0.0263 (6)	0.0291 (6)	−0.0046 (5)	0.0041 (5)	0.0024 (5)
C14	0.0305 (6)	0.0366 (7)	0.0344 (6)	−0.0081 (5)	0.0106 (5)	−0.0007 (5)
C15	0.0405 (7)	0.0263 (6)	0.0341 (7)	−0.0053 (5)	0.0114 (5)	0.0014 (5)
C16	0.0254 (6)	0.0292 (6)	0.0260 (6)	−0.0012 (4)	0.0025 (5)	0.0023 (5)
C17	0.0315 (6)	0.0243 (6)	0.0261 (6)	0.0006 (4)	0.0036 (5)	0.0032 (4)
C18	0.0286 (6)	0.0266 (6)	0.0268 (6)	−0.0008 (4)	0.0086 (5)	−0.0036 (4)
C19	0.0332 (7)	0.0377 (7)	0.0435 (7)	0.0000 (5)	0.0147 (6)	0.0070 (5)

### Geometric parameters (Å, º)

**Table:**

O1—C4	1.3701 (16)	C8—C10	1.5084 (18)
O1—H2	0.912 (17)	C8—H1c8	0.96
O2—C1	1.3715 (15)	C8—H2c8	0.96
O2—H1	0.923 (17)	C8—H3c8	0.96
N1—C9	1.4552 (17)	C9—H1c9	0.96
N1—C11	1.4863 (15)	C9—H2c9	0.96
N1—C15	1.4919 (15)	C11—H1c11	0.96
N2—C9	1.4707 (13)	C11—H2c11	0.96
N2—C14	1.4678 (17)	C12—C16	1.3924 (17)
N2—C18	1.4652 (17)	C12—H1c12	0.96
C1—C2	1.4048 (14)	C13—C16	1.3909 (17)
C1—C17	1.3852 (18)	C13—H1c13	0.96
C2—C5	1.3913 (17)	C14—C15	1.5250 (17)
C2—C11	1.5091 (18)	C14—H1c14	0.96
C3—C4	1.4022 (16)	C14—H2c14	0.96
C3—C12	1.3917 (18)	C15—H1c15	0.96
C3—C18	1.5061 (17)	C15—H2c15	0.96
C4—C7	1.3876 (18)	C16—C19	1.508 (2)
C5—C10	1.3923 (18)	C17—H1c17	0.96
C5—H1c5	0.96	C18—H1c18	0.96
C6—C10	1.3974 (15)	C18—H2c18	0.96
C6—C17	1.3884 (18)	C19—H1c19	0.96
C6—H1c6	0.96	C19—H2c19	0.96
C7—C13	1.385 (2)	C19—H3c19	0.96
C7—H1c7	0.96		
			
C4—O1—H2	106.8 (11)	C6—C10—C8	121.43 (11)
C1—O2—H1	103.3 (10)	N1—C11—C2	110.39 (9)
C9—N1—C11	112.56 (9)	N1—C11—H1c11	109.47
C9—N1—C15	103.97 (9)	N1—C11—H2c11	109.47
C11—N1—C15	111.06 (9)	C2—C11—H1c11	109.47
C9—N2—C14	102.68 (9)	C2—C11—H2c11	109.47
C9—N2—C18	114.29 (9)	H1c11—C11—H2c11	108.54
C14—N2—C18	112.76 (9)	C3—C12—C16	122.39 (11)
O2—C1—C2	120.83 (11)	C3—C12—H1c12	118.8
O2—C1—C17	118.92 (9)	C16—C12—H1c12	118.81
C2—C1—C17	120.26 (11)	C7—C13—C16	121.26 (12)
C1—C2—C5	118.38 (11)	C7—C13—H1c13	119.37
C1—C2—C11	120.41 (11)	C16—C13—H1c13	119.37
C5—C2—C11	121.20 (9)	N2—C14—C15	104.28 (9)
C4—C3—C12	118.48 (11)	N2—C14—H1c14	109.47
C4—C3—C18	120.22 (11)	N2—C14—H2c14	109.47
C12—C3—C18	121.22 (10)	C15—C14—H1c14	109.47
O1—C4—C3	121.02 (11)	C15—C14—H2c14	109.47
O1—C4—C7	119.10 (11)	H1c14—C14—H2c14	114.2
C3—C4—C7	119.88 (12)	N1—C15—C14	105.98 (11)
C2—C5—C10	122.38 (9)	N1—C15—H1c15	109.47
C2—C5—H1c5	118.81	N1—C15—H2c15	109.47
C10—C5—H1c5	118.81	C14—C15—H1c15	109.47
C10—C6—C17	121.16 (12)	C14—C15—H2c15	109.47
C10—C6—H1c6	119.42	H1c15—C15—H2c15	112.75
C17—C6—H1c6	119.42	C12—C16—C13	117.69 (12)
C4—C7—C13	120.28 (11)	C12—C16—C19	120.40 (11)
C4—C7—H1c7	119.86	C13—C16—C19	121.88 (12)
C13—C7—H1c7	119.86	C1—C17—C6	120.07 (10)
C10—C8—H1c8	109.47	C1—C17—H1c17	119.97
C10—C8—H2c8	109.47	C6—C17—H1c17	119.97
C10—C8—H3c8	109.47	N2—C18—C3	112.06 (9)
H1c8—C8—H2c8	109.47	N2—C18—H1c18	109.47
H1c8—C8—H3c8	109.47	N2—C18—H2c18	109.47
H2c8—C8—H3c8	109.47	C3—C18—H1c18	109.47
N1—C9—N2	104.65 (9)	C3—C18—H2c18	109.47
N1—C9—H1c9	109.47	H1c18—C18—H2c18	106.76
N1—C9—H2c9	109.47	C16—C19—H1c19	109.47
N2—C9—H1c9	109.47	C16—C19—H2c19	109.47
N2—C9—H2c9	109.47	C16—C19—H3c19	109.47
H1c9—C9—H2c9	113.89	H1c19—C19—H2c19	109.47
C5—C10—C6	117.74 (11)	H1c19—C19—H3c19	109.47
C5—C10—C8	120.83 (9)	H2c19—C19—H3c19	109.47

### Hydrogen-bond geometry (Å, º)

**Table:**

*D*—H···*A*	*D*—H	H···*A*	*D*···*A*	*D*—H···*A*
O1—H2···N2	0.912 (17)	1.869 (16)	2.6893 (13)	148.6 (16)
O2—H1···N1	0.923 (17)	1.825 (15)	2.6807 (12)	153.0 (15)
C17—H1*c*17···O1^i^	0.96	2.48	3.4286 (14)	168.38

Symmetry code: (i) −*x*+3/2, *y*+1/2, −*z*−1/2.

## References

[bb1] Agilent (2010). *CrysAlis PRO.* Agilent Technologies, Yarnton, England.

[bb2] Allen, F. H., Kennard, O., Watson, D. G., Brammer, L., Orpen, A. G. & Taylor, R. (1987). *J. Chem. Soc. Perkin Trans. 2*, pp. S1–19.

[bb3] Bernstein, J., Davis, R. E., Shimoni, L. & Chang, N.-L. (1995). *Angew. Chem. Int. Ed. Engl.* **34**, 1555–1573.

[bb4] Brandenburg, K. & Putz, H. (2005). *DIAMOND* Crystal Impact, Bonn, Germany.

[bb5] Cremer, D. & Pople, J. A. (1975). *J. Am. Chem. Soc.* **97**, 1354–1358.

[bb6] Palatinus, L. & Chapuis, G. (2007). *J. Appl. Cryst.* **40**, 786–790.

[bb7] Petříček, V., Dusěk, M. & Palatinus, L. (2006). *JANA2006.* Institute of Physics, Praha, Czech Republic.

[bb8] Rivera, A., Gallo, G. I., Gayón, M. E. & Joseph-Nathan, P. (1993). *Synth. Commun.* **23**, 2921–2929.

[bb9] Rivera, A., Nerio, L. S., Ríos-Motta, J., Fejfarová, K. & Dušek, M. (2012). *Acta Cryst.* E**68**, o170–o171. 10.1107/S1600536811053748 PMC3254510 22259455

[bb10] Rivera, A., Sadat-Bernal, J., Ríos-Motta, J., Pojarová, M. & Dušek, M. (2011). *Acta Cryst.* E**67**, o2581. 10.1107/S1600536811035677 PMC3201546 22065817

[bb11] Sharma, V. & Khan, M. S. Y. (2001). *Eur. J. Med. Chem.* **36**, 651–658. 10.1016/s0223-5234(01)01256-9 11600234

